# Meglumine antimoniate was associated with a higher cure rate than liposomal amphotericin B in the treatment of American tegumentary leishmaniasis: A retrospective cohort study from a *Leishmania braziliensis*-endemic area

**DOI:** 10.3389/fcimb.2022.993338

**Published:** 2022-09-23

**Authors:** Daniel Holanda Barroso, Renata Trindade Gonçalves, Joadyson Silva Barbosa, Jorgeth de Oliveira Carneiro da Motta, Gustavo Subtil Magalhães Freire, Ciro Martins Gomes, Raimunda Nonata Ribeiro Sampaio

**Affiliations:** ^1^ Hospital Universitário de Brasília, Universidade de Brasília, Brasília, Brazil; ^2^ Laboratório de Dermatomicologia da Faculdade de Medicina, Universidade de Brasília, Brasília, Brazil; ^3^ Programa de Pós-Graduação em Ciências Médicas, Faculdade de Medicina, Universidade de Brasília, Brasília, Brazil; ^4^ Pós-Graduação de Ciências da Saúde da Faculdade de Ciências Saúde, Universidade de Brasília, Brasília, Brazil

**Keywords:** therapy, liposomal amphotericin B (LAB), N-methyl glucamine antimoniate, adverse effect, American cutaneous leishmaniasis (ACL), mucosal leishmaniasis

## Abstract

**Background:**

Pentavalent antimonials (PAs) are the primary therapeutic option for American tegumentary leishmaniasis (ATL). However, the use of these drugs is complicated by adverse events (AEs), resistance and contraindications. Alternative therapies relative effectiveness is not well established.

**Objective:**

This study compared the effectiveness of liposomal amphotericin B (LAB) with intravenous meglumine antimoniate (NMG) in the treatment of ATL. We also analysed and compared associated AEs and treatment interruption rates.

**Methods:**

This was a retrospective cohort study from Brazil. The potential risk factors for the primary outcome were age, sex, total cutaneous lesion area, presence of mucosal lesions, AEs and treatment interruption. The primary outcome was lesion healing within 6 months of treatment. AEs and treatment interruption were also analysed. Multiple analytic strategies were employed to evaluate the reliability of the results.

**Results:**

Before propensity score (PS) matching, patients in the LAB group were older and had a higher frequency of mucosal lesions. The NMG group had a higher cure rate than the LAB group (cure rate 88% versus 55% respectively) in the adjusted analysis (relative risk (RR)=1.55 95% CI: 1.19 - 2.02) and after PS matching (RR=1.63 95% CI: 1.20 - 2.21). NMG group had a higher AE rate (event rate 52% versus 44%) in the adjusted analysis (RR= 1.61, 95% CI: 1.06 - 2.43, p=0.02), but this result was not observed after PS matching (RR= 0.87, 95% CI: 0.49 -1.52, p= 0.61).

**Conclusions:**

We observed that the NMG group had a higher cure rate than the LAB group, with an equivocally higher EV rate in the adjusted analysis.

## Introduction

Leishmaniasis is a vector-borne disease caused by a protozoan in the *Leishmania* genus (Burza et al., 2018); it is known to cause a wide variety of clinical syndromes, with an estimated world incidence of 700,000 to 1 million cases each year (World Health organization, 2021). The disease burden is estimated to be higher than those of leprosy, dengue fever and Chagas disease (Hotez et al., 2004). American tegumentary leishmaniasis (ATL) is likely to have a greater impact on a patient`s quality of life due to the possible development of deforming mucosal lesions (Motta et al., 2007; Luz et al., 2014).

Although the therapeutic landscape is slowly changing, pentavalent antimonials (PAs) (including N-methyl glucamine, NMG) are currently the first-line treatment for ATL (González et al., 2009; Pinart et al., 2020). The use of these drugs is problematic because they can induce severe and potentially fatal adverse events (AEs), such as arrhythmias, renal toxicity, hepatitis and pancreatitis (Kopke et al., 1993; Oliveira et al., 2011; Lyra et al., 2016). Alternatives to PAs include amphotericin B formulations, pentamidine, miltefosine, fluconazole, and ketoconazole (Aronson et al., 2017). According to a recent systematic review, however, for ATL, none of these drugs can be considered equivalent to PAs with a high or moderate level of evidence (Pinart et al., 2020). This may reflect poor designs of and reporting in most studies (Pinart et al., 2020).

Amphotericin B is an antifungal agent that has been used to treat leishmaniasis since 1960 (SAMPAIO et al., 1960); it is generally considered a second-line treatment in cases of therapeutic failure, contraindications or intolerance to PAs (Berman, 1988; Lima et al., 2007). Despite its recommendation in therapeutic guidelines (Aronson et al., 2017; Transmissíveis, 2017), published studies have shown ambiguous results regarding its efficacy (González et al., 2009; Pinart et al., 2020). Additionally, amphotericin B use has been classically associated with moderate to severe AEs, but high-quality studies evaluating this topic are scarce (Oliveira et al., 2011). Furthermore, lipid formulations of this drug, with better safety and efficacy profiles, have been studied (Walsh et al., 1999; Kleinberg, 2006; Grazziotin et al., 2018), making it challenging to derive definitive conclusions about the optimal drug for treatment. Liposomal amphotericin B (LAB) is a currently available systemic antileishmanial agent that has been successfully used in case series to treat old world cutaneous leishmaniasis due to *Leishmania major* (Wortmann et al., 2010), *Leishmania tropica* (Solomon et al., 2011) and *Leishmania aethiopica* (Zanger et al., 2011). In ATL, this drug was initially proposed in Brazil by our group with World Health Organization (WHO) sponsorship (Sampaio and Marsden, 1997), being reportedly useful to treat the main new world species: *Leishmania braziliensis, Leishmania guyanensis* (Senchyna et al., 2020), *L. amazonensis*(Soares et al., 2020) and Leishmania panamensis(Cannella et al., 2011). The liposomal formulation of amphotericin has high potential for clinical benefit due to its well-known effective management of other infections (Guery et al., 2017) and better safety profile than its conventional form with less nephrotoxicity, infusion reactions and hypomagnesemia (Wade et al., 2013). However, due to its high cost, few data from clinical studies on the clinical benefit of this formulation in the treatment of ATL are available (Guery et al., 2017). Additional challenges in the completion of clinical trials comparing amphotericin B and PAs are the high dropout and interruption rates, which are possibly related to AEs and rigorous therapeutic schedules (Neves et al., 2011; Solomon et al., 2013).

The main objective of the present study was to evaluate the ATL cure rate in patients receiving LAB and to compare the cure rate with that in those receiving intravenous meglumine antimoniate (IV-NMG) in a tertiary Brazilian leishmaniasis reference centre. We also aimed to compare the incidence rates of AEs and the rates of treatment interruption between the two treatment groups.

## Materials and methods

### Population and case definition

This retrospective cohort study included ATL patients treated with NMG or LAB at the University Hospital of Brasília, Brazil, from 1992 to 2017. Inclusion criteria was the presence of a clinical lesion compatible with ATL associated with a positive parasitological test (direct examination, culture, polymerase chain reaction or the presence of amastigotes in the histopathological exam) or at least two non-parasitological exams(serology, leishmanin skin test or compatible histopathological exam) (Gomes et al., 2014). We excluded patients who received treatment 6 months prior to the main evaluation, those with a follow-up period of less than six months. We also excluded patients in use of immunosuppressive drugs or with immunosuppressive diseases including HIV/AIDS, solid organ transplant, chronic kidney disease and cancer diagnosis. In the primary analysis one hundred and ten patients were included (63 in the NMG group and 47 in the LAB group).

### Ethics

This study was approved by the research ethics committee of the faculty of medicine of the University of Brasília, with the following CAAE 62110616.8.0000.5558. The referred committee waived the requirement to obtain informed consent since the present real-world data involves no more than minimal risk to subjects.

### Sampling

Sample size calculation was performed using Stata 17 software (College Station, TX: Stata Press. StataCorp, 2021) considering the response rates of 81% in the LAB group and 99.9% in the NMG group obtained in a previous pilot study (Motta and Sampaio, 2012). Based on these rates, a sample of 37 patients in each group would result in 80% power to identify significant differences between the groups, with a significance level of 5%. Additional evaluations including the analysis of other outcomes and the analysis of simultaneous predictors were accessed by a *post hoc* strategy.

### Intervention

We compared the NMG and LAB interventions. NMG was used in accordance with the recommendations of Brazil`s Ministry of Health (10 to 20 mg SbV/kg/day for 20 days for the cutaneous form and for 30 days for the mucosal form). LAB was administered at a dosage of 1-3 mg/kg/day in at least 5 days.

### Outcomes

The main outcome was cure, defined as complete healing (reepithelization without infiltrations or erythema) of the lesion by the 180^th^ day after the first medication dose. Interruption of treatment for more than 7 days and AEs of any grade were secondary outcomes. According to the institutional protocol, patients were monitored at least weekly during treatment and at 2, 3 and 6 months after treatment. Laboratorial alterations in electrocardiogram results, liver enzyme levels or kidney function indicators were monitored at each visit to monitor for AEs.

### Statistical analysis

The cure rate, occurrence of AEs and treatment interruption rate were individually considered dependent variables, and the treatment group (NMG or LAB) was considered an independent variable. Initially, we performed univariate analyses to identify associations between the independent and dependent variables. Sensitivity analysis was performed to evaluate whether methodological shortcomings could be responsible for the identified associations. To evaluate whether patient characteristics associated with the intervention allocation or with the outcome could be responsible for the results, Poisson regression with robust variance was performed to obtain adjusted relative risks (RRs) based on sex (male; female), age (years), presence of mucosal lesions and total area of lesions, including no cutaneous lesions. To evaluate the outcome of cure, we added treatment interruption and AE rates into the model. To evaluate the outcome of treatment interruption, the presence of AEs was also added. To further evaluate cure, patients were matched in a 1:1 ratio based on propensity scores (PS) using the “greedy” strategy considering a calibration of 0.2 standard deviations (SDs) using the same variables analysed in the multivariate analysis. Univariate analysis of the associations of predictors variables using Poisson regression with robust variance were also done in the whole population. To evaluate whether LAB dosage variation could explain the cure rates observed in this group, a univariate Poisson regression with robust variance model was constructed considering cure as a dependent variable and LAB dosage as the independent variable. Statistical analysis was performed in SAS 9.4 (SAS Institute, Cary, NC) and Stata 17 (StataCorp, College Station, TX). The results were considered statistically significant if p<0.05.

## Results

The NMG group received the standard dosage recommended by the Brazilian Ministry of Health (15 mg SbV/kg/day for 20 days if there was no mucosal disease or for 30 days if there was mucosal disease). The total LAB dosage administered was 21.61 mg/kg ± 17.37 (SD). Patients who received LAB were older, had a higher frequency of mucosal lesions and had a lower cure rate than patients who received NMG (**Tables 1**, **2**).

**Table 1:** Sample characteristics stratified by drug before and after PS matching.

	Before PS matching			After PS matching		
Characteristic ^*^	**NMG (n = 63)**	**LAB (n = 47)**	**p value**	**NMG (n = 33)**	**LAB (n = 33)**	**p value**
**Age**	37.19 ± 18.68	51.70 ± 22.18	<0.0001^#^	41.82 ± 20.81	46.03 ± 24.14	0.4506^#^
**Total area of cutaneous lesions**	11.24 ± 17.51	9.37 ± 13.52	0.3130†	13.60 ± 21.46	8.57 ± 10.20	0.4649†
**Sex**						0.2284^ƍ^
Female	17 (26.98)	12 (25.53)	0.8642 ^ƍ^	5 (15.15)	9 (27.27)	
Male	46 (73.02)	35 (74.47)		28 (84.85)	24 (72.73)	
**Mucosal lesions**						0.2840^ƍ^
No	45 (71.43)	25 (53.19)	0.0492 ^ƍ^	25 (75.76)	21 (63.64)	
Yes	18 (28.57)	22 (46.81)		8 (24.24)	12 (36.36)	

* values expressed as the mean ± standard deviation or frequency (%).

Univariate analysis using #Student’s T test, (†)Mann−Whitney or ƍ chi-square test.

NMG, meglumine antimoniate; LAB, liposomal amphotericin B; PS, propensity score; n, number of patients.

**Table 2:** Outcomes before and after PS matching.

	Before PS matching			After PS Matching			
Outcome^*^	NMG (n = 63)	LAB (n = 47)	p value^#^	NMG (n = 33)	LAB (n = 33)	p value^#^	
**Interruption**						0.7412	
No	54 (85.71)	34 (72.34)	0.0828	27 (81.82)	28 (84.85)		
Yes	9 (14.29)	13 (27.66)		6 (18.18)	5 (15.15)		
**Adverse events**						0.6184	
No	30 (47.62)	26 (55.32)	0.4242	20 (60.61)	18 (54.55)		
Yes	33 (52.38)	21 (44.68)		13 (39.39)	15 (45.45)		
**Cure**						0.0006	
No	7 (11.11)	21 (44.68)	<0.0001	2 (6.06)	14 (42.42)		
Yes	56 (88.89)	26 (55.32)		31 (93.94)	19 (57.58)		

* values expressed as the mean ± standard deviation or frequency (%).

P value calculated using chi-square test.

NMG, meglumine antimoniate; LAB, liposomal amphotericin; B, PS, propensity score; n, number of patients.

**Figure 1:**
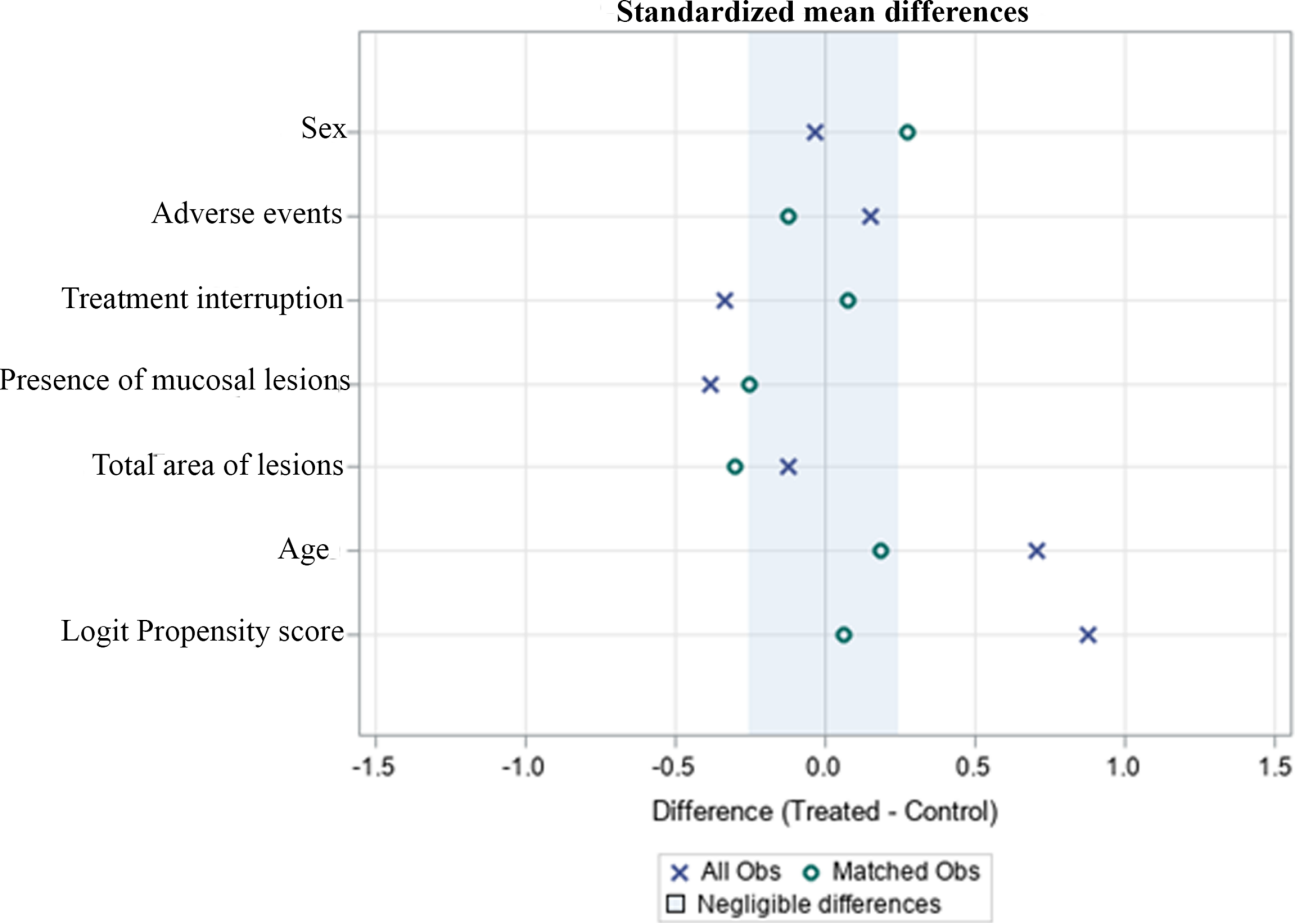
Standardized differences before and after PS matching comparing variables for patients treated with NMG and LAB drugs.

We were able to match 33 patients in each treatment arm based on their PSs, obtaining well-balanced groups (**Figure 1**). The NMG group had a higher cure rate than the LAB group (cure rate 88% versus 55% respectively) in the adjusted analysis (RR=1.55 95% CI: 1.19 - 2.02) and after PS matching (RR=1.63 95% CI: 1.20 - 2.21). NMG group had a higher AEs rate (event rate 52% versus 44%) in the adjusted analysis (RR= 1.61, 95% CI: 1.06 - 2.43, p=0.02), but this result was not observed after PS matching (RR= 0.87, 95% CI: 0.49 -1.52, p= 0.61) (**Table 3**).

**Table 3:** Unadjusted and adjusted relative risks (RRs) and 95% confidence intervals (CIs) for cure, interruption and AEs before and after propensity score matching.

					Before PS matching					After PS matching		
Outcome	Drug	RR (95% CI)			p value		Adjusted RR^*^ (95% CI)	p value		RR (95% CI)	p value
Cure	**NMG**	1.61 (1.22; 2.11)			0.0006	1.55 (1.19; 2.02)		0.0013		1.63 (1.20; 2.21)	0.0017
Interruption	**NMG**	0.52 (0.24; 1.11)			0.0890	0.60 (0.30; 1.19)		0.1401		1.20 (0.41; 3.55)	0.7417
Adverse events	**NMG**	1.17 (0.79; 1.74)			0.4310	1.61 (1.06; 2.43)		0.0252		0.87 (0.49; 1.52)	0.6194

* For all the outcomes, the relative risks was adjusted by the variables in **Table 1** using Poisson regression analysis. For the outcome cure, interruption and AEs were added to the model, and for the outcome interruption, AEs were added to the model.

RR, Relative risk; 95% CI, 95% confidence interval; PS, Propensity score; NMG, meglumine antimoniate.

The LAB dosage was not associated with cure in the dosage range applied in this study. In the whole population, we found significant association between age 60 or greater and the outcome interruption of treatment (RR= 3.68, 95% CI: 1.75-7.73, p<0.01) and adverse events (RR= 1.75, 95% CI: 1.23- 2.48, p<0.01). Other relevant influences on the tested outcomes were not detected.

## Discussion

High-quality clinical trials comparing the use of PAs with amphotericin B in ATL patients are lacking, and the recommendations of health agencies are based on case series and retrospective studies (Aronson et al., 2016; Pinart et al., 2020). The reported efficacy of amphotericin B in the literature is greater than 90% (Sampaio et al., 1971; Wortmann et al., 2010; Amato et al., 2011; Rocio et al., 2014). In a study from Bolivia, LAB had a superior cure rate when compared with sodium stibogluconate (SSG) (84% versus 70%), but the results were nonsignificant (Solomon et al., 2013). We expected that increasing the sample size would lead to significant differences between the groups. The better effectiveness of NMG observed in our study is not surprising since this is the standard drug for ATL treatment and the treatment with which other treatments are compared (Pinart et al., 2020). A study from French Guyana also showed a lower cure rate in patients who received LAB than in those who received NMG (Senchyna et al., 2020), although the difference did not reach statistical significance. The overall cure rate in LAB patients in this study (55.32%) was similar to that in the study by Guery et al. (44%), which also included patients with Old-World leishmaniasis (Guery et al., 2017). In another case series that included only mucosal leishmaniasis patients from Brazil, the cure rate was 93.1% (Cunha et al., 2015). This difference may be explained by the fact that in their study, therapeutic failure was defined as the absence of clinical response after two successive therapeutic cycles (Cunha et al., 2015), whereas retreatment with the same therapeutic scheme has been reported to promote clinical cure in some patients (Nogueira and Sampaio, 2001).

We also investigated the associations between treatment interruption and AEs. A previous study showed a higher rate of treatment interruption in patients who received PAs (SSG) than in those who received LAB (Solomon et al., 2013). In the study by Senchyna et al., NMG was associated with a higher rate of moderate AEs, defined as those with clinical symptoms but that did not lead to treatment interruption (Senchyna et al., 2020). Despite the possible development of formulation-specific adverse reactions (Szebeni et al., 2000; Roden et al., 2003), LAB is known to have a better safety profile than the other formulations (Wasan et al., 1994; Walsh et al., 1999; Wade et al., 2013). Accordingly, in this study, the adjusted RR for adverse events was higher in patients who received NMG than patients who received LAB. Although this result reached statistical significance, it was not reproduced after matching. Thus, the higher AE rate in the NMG group should be interpreted with caution and deserves further evaluation in larger studies specifically powered to evaluate comparative AEs between medication groups.

The age of patients and proportion of mucosal lesions in the current study are likely to be different from those in the overall population of ATL patients since older people and those with mucosal lesions are more likely to be referred to a tertiary care centre. In an epidemiological study performed in a primary care setting in Bahia, Brazil, only 4.3% of patients had mucosal lesions, and the average age of cutaneous leishmaniasis patients was 21 years (Jirmanus et al., 2012), which was younger than 37 years (NMG group) and 51 years (LAB group), as reported in our study. Thus, the convenience sample used in this study may limit the generalizability of our results. Additionally, in the primary analysis, patients who received LAB were significantly older and had a significantly higher frequency of mucosal lesions. Again, this may be explained by the increased risk of mucosal lesions with age (Machado-Coelho et al., 2005) and by the recommendation that people aged 50 years or older should be treated with amphotericin B according to the national guidelines(Transmissíveis, 2017). The lower cure rate observed in patients who received LAB, however, is unlikely explained by their basic characteristics since these results were consistent across multiple analytic strategies that included controlling for confounders.

One of the limitations of this study is its observational design. Although randomized clinical trials (RCTs) are the gold standard for analysing the intended effects of therapies, observational studies are as valid as RCTs to investigate AEs associated with medications (Vandenbroucke, 2008).Additionally, Interruption and dropout rates can be a treat to internal validity of clinical trials (Ravani et al., 2007) and have been important in studies of LAB for the treatment of leishmaniasis (Neves et al., 2011; Solomon et al., 2013). LAB studies have been limited by the cost of the medication, especially considering that ATL is highly prevalent in low-income countries (Guery et al., 2017). Thus, the relatively low cost, wide range of patients and rapidly obtained conclusions make observational studies an interesting approach to investigate the effects of LAB in ATL patients (Benson and Hartz, 2000). The main limitation of observational studies is related to treatment allocation, but the strategy used by our team, propensity analysis, is known to offset this issue (Feneck, 2007). Provided that important confounders are controlled for (Vandenbroucke, 2008), it has been shown that observational studies can produce results similar to those of RCTs (Benson and Hartz, 2000).

We therefore attempted to overcome the limitations of previous studies using an observational design coupled with an adequate analytical strategy. To do this we performed *post hoc* adjusted and PS matched analysis including clinical and individual characteristics to try to explain the associations found in the unadjusted analysis. As shown by other studies, age is associated with increased adverse events rate (Araujo-Melo et al., 2010; Diniz et al., 2012; do Lago et al., 2018) and immunological responses (Carvalho et al., 2015). As expected, we have found that elderly patients had a higher rate of adverse events and interruption of treatment but, as previously suggested, we were not able to find an association between age and treatment response (do Lago et al., 2018). Although sex (de Araújo Albuquerque et al., 2021), total area of lesions (Valencia et al., 2012) and the presence of mucosal lesions (García-Bustos et al., 2021) were all previously related with treatment failure, we were not able to find significant relationship with the outcome in our data.

In this cohort study from a *L. braziliensis-*endemic area (Gomes, 2014), NMG was associated with a higher cure rate than LAB, although it also had an equivocally higher AE rate. The consistency of the primary results across multiple analysis and their applicability in the real world setting of a Brazilian reference centre are the main strengths of this study. Is important to state, however, that their validity in the overall population is limited. Possible known confounders were controlled for in the analysis, but the presence of unknown covariates is a limitation in any observational study. As randomization is the only way to balance these covariates, our results should be confirmed in a large RCT.

## Data availability statement

The raw data supporting the conclusions of this article will be made available by the authors, without undue reservation.

## Ethics statement

The studies involving human participants were reviewed and approved by Comitê de Ética em Pesquisa da Faculdade de Medicina da Universidade de Brasília, CAAE 62110616.8.0000.5558. Written informed consent for participation was not provided by the participants’ legal guardians/next of kin because: The referred committee waived the requirement to obtain informed consent since the present real-world data involves no more than minimal risk to subjects.

## Author contributions

DB – conception, design, data acquisition, analysis, interpretation of data, drafting; RG - data acquisition, analysis, interpretation of data; JB - data acquisition, analysis, interpretation of data; JM - data acquisition, analysis; GM- data acquisition, analysis and interpretation; CG - conception, design, data acquisition, analysis, interpretation of data, drafting; RS - conception, design, data acquisition, analysis, interpretation of data, drafting, supervision. All: Final approval.

## Conflict of interest

The authors declare that the research was conducted in the absence of any commercial or financial relationships that could be construed as a potential conflict of interest.

## Publisher’s note

All claims expressed in this article are solely those of the authors and do not necessarily represent those of their affiliated organizations, or those of the publisher, the editors and the reviewers. Any product that may be evaluated in this article, or claim that may be made by its manufacturer, is not guaranteed or endorsed by the publisher.
